# Effect of Academic Self-Efficacy and Career Decision Level on Career Preparation Behavior of South Korean College Students

**DOI:** 10.3390/ijerph192013705

**Published:** 2022-10-21

**Authors:** Hyemin Kim, Young-An Ra

**Affiliations:** School of Counseling Psychology and Social Welfare, Handong Global University, Pohang-si 37554, Korea

**Keywords:** academic self-efficacy, career decision level, career preparation behavior

## Abstract

The purpose of this study was to examine the mediating role of the career decision level (decidedness and comfort) of South Korean college students regarding the relationships between academic self-efficacy and career preparation behavior. For this purpose, the study collected and analyzed data on 296 South Korean college students using SPSS 22.0 and Hayes’ (2013) Process Macro tool. The results of the study are as follows. Academic self-efficacy was positively associated with career decision level and career preparation behavior, and career decision level was positively associated with career preparation behavior. In terms of the mediation effect, the career decision level partially mediated the relationships between academic self-efficacy and career preparation behavior. The results of the study provide useful suggestions for interventions in the career counseling of college students.

## 1. Introduction

Recently, many college students have been struggling with anxiety about their careers [1]. According to Han [2], only 20 percent of college students reported that they had chosen their career path, while 40 percent of students were still wondering which career to choose. As this study has shown, college students are facing many difficulties and worries regarding their career development. This reality calls for an urgent need to help them prepare for future careers. However, career education and counseling for college students mainly aim to increase the employment rate, without providing sufficient opportunities to explore one’s career interests and aptitudes. This has made it difficult for students to fully explore and understand the kind of career path that they want to pursue [3].

Proper career preparation is an important developmental task for college students. According to Super [4], college students are in the stage of exploration and growth, in which they can understand themselves through practical experiences in school and leisure activities. Additionally, being in college is an opportunity to gain professional experience before they enter the workforce [5]. College students start to compromise their career choice by considering personal needs and interests, values, and practical aspects of their professional abilities and career opportunities. Students can prepare for their careers through various experiences, including participating in job interviews, writing resumes, consulting a career center, and participating in career counseling and coaching [5].

However, Park and Lee [6] noted that South Korean college students are good at studying but do not have enough time to prepare for their careers. Korean society or parents force students to achieve high grades in college rather than searching for their career interests or aptitudes [7]. Since many South Koreans college students are facing these career difficulties, more studies are needed to help these populations to increase their career preparations. Then, we can ask what factors can empower the preparation of a career? The performance model of Social Cognitive Career Theory (SCCT) indicates that self-efficacy can directly influence career performance and indirectly influence performance level by setting a performance goal [8]. Self-efficacy is a person’s belief that they have an ability to organize and execute the actions required to manage prospective situations [9]. Successful experience can reinforce the formation of self-efficacy, and the self-efficacy of college students is closely related to their academic performances [9]. Many scholars have reported that self-efficacy is also closely related to career preparation behavior [10]. Academic self-efficacy is especially essential for South Korean students. The relationships between college students’ academic self-efficacy and their career developments have thus been proven by previous research [11]. In addition, Lent and his colleagues [8] suggested that when an individual decides which career to pursue and has a specific motivation to make a decision, they may begin to have career aspirations. Choosing a specific occupation or career could be a concrete goal to pursue. Thus, career decisions can correspond to the concept of career goals in SCCT. In fact, college students are usually at this stage before entering the workforce, and they need to make a practical decision. Thus, career decision is another important factor that has an impact on career preparation. Moreover, previous research has suggested that career decision level and academic self-efficacy are significantly interrelated. Kim [12] asserted that academic self-efficacy is an essential variable in predicting stable career development, and career decision level and academic self-efficacy show a significant positive correlation [13]. The studies that examined the relationships between academic self-efficacy, career stress, and career decision level showed that academic self-efficacy significantly predicted an increase in career decision level [12]. Thus, when students have a positive belief about academic performance, they tend to have a firm career decision as a concrete goal to pursue.

In summary, the purpose of the present study is to examine the relationships between career preparation, academic self-efficacy and career decision, which are important in the career development of South Korean college students. Since there are few studies that focused on these three variables, especially based on the theoretical assumptions from SCCT, the present study is able to provide a novel contribution to the career-related literature. Therefore, these are the research questions of the study: What are the relationships between academic self-efficacy, career decision level (decidedness and comfort) and career preparation behavior? Does career decision level (decidedness and comfort) mediate the relationship between academic self-efficacy and career preparation behavior?

## 2. Methodology

### 2.1. Participants

To calculate an appropriate sample size, we employed Tabachnick and Fidell’s [14] formula *n* ≥ 50 + 8 m, where m is equal to the number of variables. The participants of the present study were 296 undergraduate students from a 4-year college in South Korea. The study sample consisted of men (39.3%, *n* = 118), and women (60.7%, *n* = 182) with an average age of 23.07 years (age range = 18~33 years, SD = 2.56).

### 2.2. Measures

#### 2.2.1. Academic Self-Efficacy

We used the South Korean version of the academic self-efficacy scale developed by Kim and Park [15]. This measures an individual’s confidence in their academic performance. The scale consisted of 28 items, each rated on a 6-point Likert scale (1 = strongly disagree, 6 = strongly agree). Sample items of the scale are “I can concentrate well even when I don’t like the lecture”, and “I enjoy the process of solving a complex and difficult problem”. Cronbach’s alpha values of this measurement in Kim and Park’s [15] study were from 0.74 to 0.84. In the present study, the internal consistency was 0.90. 

#### 2.2.2. Career Preparation Behavior

We used the career preparation behavior scale developed by Kim [16] and revised by Lee [17]. The scale consisted of 18 items, each rated on a 4-point Likert scale (1 = strongly disagree, 4 = strongly agree). The sample items are “In the last few weeks, I had a talk with my friends about my interest and my future career (job)” and “In the last few months, I had visited an institution or made a plan to visit an organization that is related to career or job that I am interested in”. Lee’s study [17] reported 0.88 for Cronbach’s alpha of the measurement. The present study reported 0.93 of internal consistency.

#### 2.2.3. Career Decision Level

We used Career Decision Profile (CDP) to measure decidedness and comfort levels. This provides an estimate of career indecision and its effects of relevant decision. The scale was invented by Jones [18]. We used Korean version of CDP developed by Gao [19]. The scale consisted of two subscales (decidedness and comfort). and4 items and was rated on a 4-point Likert scale (1 = strongly disagree, 4 = strongly agree). The sample item of decidedness was “I have an occupational field in mind that I want to work in,” and the sample item of comfort was “I feel at ease and comfortable with where I am in making a vocational decision”. Cronbach’s alpha of the measurement in Gao’s study [19] was 0.82. In the present study, Cronbach’s alpha of decidedness was 0.84 and comfort was 0.81.

#### 2.2.4. Demographic Information

We controlled the individual variables (gender, major, grade and GPA) that are found to have a significant influence on career preparation behavior [20]. The study thus coded engineering, natural science, and medical science as a STEM group, and humanities, social sciences, business, education, music, art, and sports as a non-STEM group according to previous studies [21].

### 2.3. Procedure and Data Analysis

We gathered participants using an online questionnaire and they voluntarily answered the survey. We utilized SPSS 22.0 and Process Macro [22] to analyze the data, including SPSS missing-data mechanism, which was used to precisely handle the missing data. Hayes [22] developed the mediation macro model to estimate the total effect, direct effect and indirect effect at one step with bias-corrected bootstrap confidence intervals. In this study, we tested the indirect effect with 5000 bootstrappings to confirm the existence of a mediation effect, controlling demographic factors (gender, grade, majors, and GPA). In addition, we put two mediators in a single model to examine each of the indirect effects.

## 3. Results

We conducted a descriptive analysis and Pearson correlation analysis to understand the general features of the variables of interest and demographic information. The mean of academic self-efficacy was 101.17 (*SD*: 15.13), career preparation behavior was 44.01 (*SD*: 10.47, decidedness was 5.73 (*SD*: 1.49), and comfort was 4.56 (*SD*: 1.68). Academic self-efficacy weakly to moderately correlated with career preparation behavior (*r* = 0.37), decidedness (*r* = 0.21), and comfort (*r* = 0.35). Career preparation behavior moderately correlated with decidedness (*r* = 0.43) and comfort (*r* = 0.40). All correlations are statistically significant (*p* < 0.01). Based on previous research [21,22], we put decidedness and comfort together in Model 4 of the process macro with control variables of gender, grade, majors and GPA. Firstly, the total effect of academic self-efficacy on career preparation behavior was significant (*b* = 0.26, *t* = 6.32, *p* < 0.001), which means that academic self-efficacy alone significantly predicted career preparation behavior. The direct effect of academic self-efficacy on career preparation behavior was also significant (*b* = 0.15, *t* = 3.74, *p* < 0.001), meaning that even when decidedness and comfort are in the model, academic self-efficacy significantly predicts career preparation behavior. Secondly, we examined the mediating role of decidedness. Academic self-efficacy was significantly associated with decidedness (*b* = 0.04, *t* = 5.77, *p* < 0.001), and decidedness significantly predicted career preparation behavior (*b* = 1.94, *t* = 4.53, *p* < 0.001). The indirect effect of decidedness was 0.07. If the confidence interval generated from 5000 bootstrapped samples does not include zero, the indirect effect is significant at *p* < 0.05. Decidedness was found to have an indirect effect (*SE* = 0.02, CI = 0.03~0.11). Thirdly, the result of comfort’s mediating role was as follows. Academic self-efficacy had a significant influence on comfort (*b* = 0.04, *t* = 5.58, *p* < 0.001), and comfort was significantly associated with career preparation behavior (*b* = 1.13, *t* = 2.91, *p* < 0.05). The indirect effect of comfort is 0.04, and the confidence interval does not contain zero (*SE* = 0.02, CI = 0.01~0.08). Lastly, the total indirect effect of decidedness and comfort was significant (*b* = 0.11, *SE* = 0.03, CI = 0.07~0.16). In a nutshell, decidedness and comfort partially mediated the relationship between academic self-efficacy and career preparation behavior.

## 4. Discussion

We examined the relationships between the academic self-efficacy, career decision level (decidedness and comfort), and career preparation behavior of South Korean college students. We also verified the mediating effect of decidedness and comfort on the relationship between academic self-efficacy and career preparation behavior. The results for academic self-efficacy and career preparation behavior show a positive correlation, and this indicates that students with a strong academic self-efficacy in their studies tend to have a higher level of career preparedness. According to Bandura’s [9] theory, self-efficacy has a characteristic of generality, meaning that the efficacy of an individual in an academic setting can be applied to the individual’s career. The results are consistent with previous research findings [23]. Next, academic self-efficacy showed a positive association with decidedness as well as comfort, which suggests that individuals with a high academic efficacy tend to make more solid career decisions and be comfortable about their career choice. Furthermore, the results indicate that higher academic self-efficacy can support decision-making processes and empower students to set a career goal. This means that if an individual has a higher level of academic self-efficacy, they are able to make better career decisions. The results for self-efficacy and career decision making are also consistent with previous studies [20]. Lastly, career preparation behavior and career decision level also showed positive relations, echoing prior findings [6].

The second purpose of the study was to examine the mediating role of decidedness and comfort on the relationships between academic self-efficacy and career preparation behavior. We found that academic self-efficacy significantly predicted career preparation behavior, and decidedness and comfort partially mediated the association between academic self-efficacy and career preparation behavior. The demographic variables did not show any significant effects in the model. Compared to other countries, South Korean society tends to have a strong focus on educational backgrounds; most career education thus focuses on academic success [24]. For this reason, most students start shaping their career interests after entering college. Within four years of college education, they have to acquire the necessary professional knowledge and practical skills, on top of completing their coursework. The results of the present study imply that the academic self-efficacy makes these students set effective career goals, which in turn leads to higher levels of career preparation behavior. This result also reflects a previous finding: concrete careers goals motivate students to study [25]. Students with higher self-efficacy tend to make more rational decisions [9] and their high academic self-efficacy leads to less career-related stress. The results indicate that a higher academic self-efficacy should be transferred to make a firm and secure career decision in order to go through a stable preparation process.

There are significant findings from this study that can be applied in practical fields. First, practitioners and counselors working with college students should be aware of educational and academic background in career counseling sessions. Since academic self-efficacy has a strong influence on career preparation behavior, counselors need to assess the academic ability, learning strategy, and academic motivation of their clients and understand their educational backgrounds, especially when they are dealing with South Korean college students. They should also be able to develop many activities and resources to increase students’ self-efficacy such as worksheets for self-esteem and self-talk. In doing so, students’ academic self-efficacy may increase, causing an increase in career decision level and career preparation behavior. Secondly, institutions such as a student counseling center, career development center, or education development center in a university or college could consider how to connect academic abilities when developing a career preparation program.

There are some limitations to this study. First, we collected data by online survey. The online surveys are advantageous since respondents can easily and quickly respond to the survey; however, it is difficult to control dishonest response. Additionally, data were collected by a self-reporting questionnaire, so the actual levels of main constructs may differ from the scores of the scale.

## 5. Conclusions

In conclusion, the results of the present study show that the career decision levels of decidedness and comfort partially mediated the relationships between academic self-efficacy and career preparation in South Korean college students. Based on the results, higher education institutions could develop college career development programs that focus on students’ levels of decidedness and comfort regarding career decision, developing activities that can stimulate academic abilities and those career decision levels to ultimately promote their career preparation behaviors. 

## Data Availability

The data that were used in the present study can be provided by the corresponding author upon reasonable request.
